# Antimicrobial Properties of Monomeric and Dimeric Catanionic Surfactant System

**DOI:** 10.3390/molecules30010164

**Published:** 2025-01-03

**Authors:** Iwona Kowalczyk, Anna Koziróg, Adrianna Szulc, Anna Komasa, Bogumił Brycki

**Affiliations:** 1 Department of Bioactive Products, Faculty of Chemistry, Adam Mickiewicz University Poznan, 61-614 Poznan, Poland; iwkow@amu.edu.pl (I.K.); adrszu@gmail.com (A.S.); aniak@amu.edu.pl (A.K.); 2 Institute of Fermentation Technology and Microbiology, Faculty of Biotechnology and Food Science, Lodz University of Technology, 90-924 Lodz, Poland; anna.kozirog@p.lodz.pl

**Keywords:** catanionic surfactant system, antibacterial activity, DFT calculations

## Abstract

Cationic gemini surfactants are used due to their broad spectrum of activity, especially surface, anticorrosive and antimicrobial properties. Mixtures of cationic and anionic surfactants are also increasingly described. In order to investigate the effect of anionic additive on antimicrobial activity, experimental studies were carried out to obtain MIC (minimal inhibitory concentration) against *E. coli* and *S. aureus* bacteria. Two gemini surfactants (12-6-12 and 12-O-12) and two single quaternary ammonium salts (DTAB and DDAC) were analyzed. The most commonly used commercial compounds of this class, i.e., SDS and SL, were used as anionic additives. In addition, computer quantum mechanical studies were also carried out to confirm the relationship between the structure of the mixture and the activity. The obtained results of microbiological tests and quantum mechanical calculations are in agreement with each other and show the lack of synergism in catanionic mixtures in the case of antibacterial activity.

## 1. Introduction

Surfactants are essential elements in various, complex industrial applications, in processes and in consumer products. First of all, by lowering the surface tension of aqueous solutions, they can serve as cleaning, foaming or wetting agents, as well as dispersants, solubilizers, emulsifiers, demulsifiers or additives and viscosity modifiers [1,2,3,4]. Moreover, thanks to the ability to accumulate at the interface, they exhibit antimicrobial, antistatic or anticorrosive properties. Surfactant blends can form mixed micellar aggregates that are usually superior in achieving better technical and economic advantages than those of individual components. These properties make surfactant substances that are used in almost every area of life and industry [1,5,6,7,8,9,10]. The innovative applications of surfactants in medicine or pharmacology are also worth special attention. It is estimated that the global annual production of surfactants reached USD 41.84 billion in 2022. It is also predicted to increase to about USD 60 billion by 2030 [11].

Over the past few decades, significant efforts have been made to create new surface-active agents called gemini. Compared to conventional single-chain surfactants, gemini surfactants use spacer groups to connect two single-chain surfactant molecules [12,13,14,15]. The most commonly obtained and used dimeric surfactants are double quaternary ammonium salts. The unique molecular structure reinforces the interaction between the hydrophobic chains and relieves the repulsive force between the hydrophilic groups; therefore, gemini surfactants exhibit excellent surface/interfacial properties [16,17,18,19].

A catanionic surfactant (i.e., a mixture of cationic and anionic surfactants) is a new solution in the world of surfactants [20,21,22,23]. To compare the surface activity of various substances, the most useful parameter is critical micelle concentration (CMC). Catanionic surfactants belong to the class of mixtures with extremely low CMC, which is one or two orders of magnitude lower than single ionic surfactants [24]. The development of this class of surfactants undoubtedly began with Kaler’s publication [25]. Since then, considerable efforts have been made to study the self-assembling behavior of catanionic surfactants. Initially, only systems consisting of single cationic surfactants mixed with anionic ones were described. Currently, much attention is focused on systems composed of double cationic surfactants with the addition of anionic surfactants [26,27]. It is worth mentioning that in the case of surface activity, catanionic surfactants show a synergistic effect. They exhibit significantly lower critical micelle concentration, faster diffusion rates, and superior wetting capability compared to single surfactants. The micellization process of both mixed systems is spontaneously enthalpy-driven, and the diffusion–adsorption process is consistent with the mixed diffusion–kinetic mechanism [27]. Since interactions between ionic surfactants are caused by the electrostatic forces between their head groups, it will be expected that such interaction would be stronger for surfactants having two ionic groups [28,29,30]. Binary mixtures of gemini surfactants with single-tail anionic surfactants are more likely to exhibit synergism, although the degree of synergism is caused by the head group variations as well as chain length variations. Wang et al. reported that surfactant mixtures not only reduce the application costs by decreasing the amount of gemini surfactant but also improve the performance of the surfactant through synergy between different surfactants [31]. Owing to the various applications and differences in properties of mixed systems and individual components, research of mixed systems of gemini and conventional surfactants must be very important for both practical and fundamental aspects as their properties will be dictated by their aggregation behavior and composition [28].

The most useful property of gemini surfactants is their antimicrobial activity. These compounds exhibit biocidal activity against a wide range of microorganisms: Gram-positive and Gram-negative bacteria, yeasts and molds [32,33,34,35,36,37]. Recent literature reports indicate the antiviral activity of these compounds [38].

This article is a response to the very small number of reports on the effect of anionic surfactant addition on the antimicrobial activity of a catanionic system. The novelty of the study compared to the previous literature is the determination of the effect of anionic additives on the antibacterial activity of double and single quaternary ammonium salts. For this reason, in this article, we present conclusions on how the addition of an anionic surfactant affects the antibacterial activity of single and double ammonium salts. We also attempted to explain this phenomenon using DFT calculations.

## 2. Results and Discussion

### 2.1. Preparation of Solutions of Catanionic Mixtures

**Figure 1:**
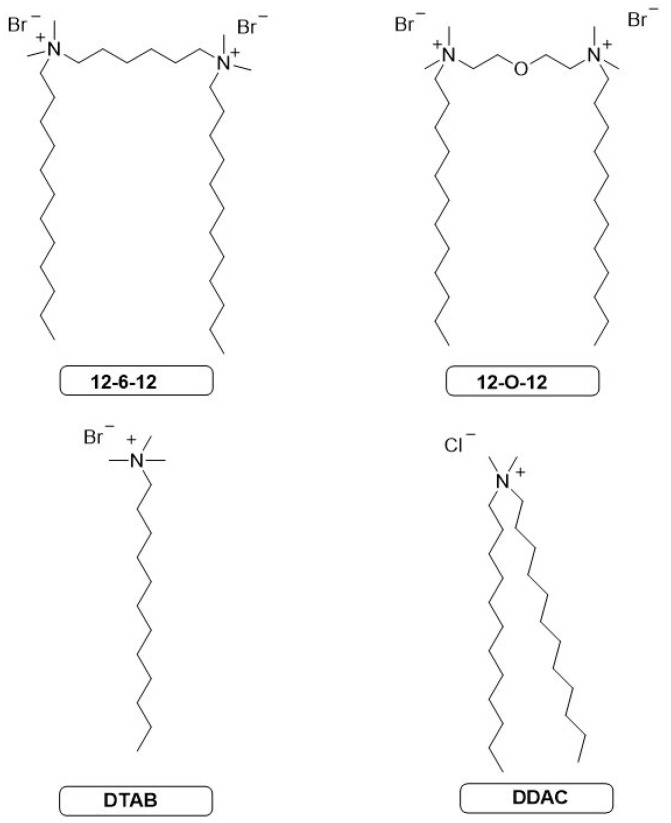
Structures and abbreviations of cationic surfactants.

We obtained eight series of mixtures of catanionic surfactants. Gemini surfactants were obtained in our laboratory from a preparation recipe consistent with the previously published one, [11] for 12-6-12 and [39] for 12-O-12. The single quaternary ammonium salts used to prepare the mixtures are commercially available. The formulas and abbreviations of the single and double quaternary ammonium salts used are presented in Figure 1.

In order to obtain catanionic mixtures, we used sodium dodecyl sulfate (SDS) and sodium dodecanoate (SL). The initial surfactant concentration was 1 mM. Detailed amounts of individual surfactants used to prepare the mixtures are given in Table 1.

### 2.2. Antimicrobial Activity

The difference in the mechanisms of action of cationic as well as anionic surfactants on Gram-negative and Gram-positive bacteria at the molecular level is mainly due to differences in the structure and composition of their cell membranes, including the presence of an outer cell membrane in Gram-negative bacteria and a thick cell wall in Gram-positive bacteria.

The cell wall of Gram-positive bacteria is much thicker than that of Gram-negative bacteria, and its structure contains teichoic acids. The forces of electrostatic attraction determine the binding of positively charged surfactants to the available negatively charged phosphate groups in the teichoic acid chains. This bonding leads to a reduction in cell wall stability and affects its integrity. The change in cell wall structure can weaken bacteria, making them more susceptible to external agents, including other chemicals. Cationic surfactants can also affect proteins in the membrane, which have transport functions or are involved in signal transduction, among other things. Damage to proteins through electrostatic interactions often leads to destabilization of the membrane structure and increases its permeability. The membrane also contains phospholipids, which are its main component. Interaction of these negatively charged compounds with positively charged surfactant groups can destabilize the lipid bilayer, resulting in increased membrane permeability and even cell lysis. Changes in the cytoplasmic membrane affect ion transport, and as a result, membrane potential and energy production are disrupted.

The action of cationic surfactants on Gram-negative bacteria, on the other hand, at the cellular level is more complex than that of Gram-positive bacteria, due to the presence of an outer membrane that surrounds the cell and provides an additional barrier. This membrane contains lipopolysaccharides (LPSs) consisting of a lipid (lipid A) and a polysaccharide part. Cationic surfactants can interact with negatively charged phosphate groups in the lipid part of the LPSs, which contributes to a change in the external structure. Subsequently, after destabilization of the membrane, these compounds can reach the inner membrane, causing disruption of its structure and increasing permeability. With membrane damage, membrane proteins are disrupted, and ion transport and cellular metabolism are disrupted. The result of this action is an increase in membrane permeability, a loss of ionic homeostasis and, consequently, the death of the bacterial cell [40,41,42,43].

Cationic surfactants are more effective in destroying bacteria, especially Gram-positive bacteria, while anionic surfactants show weaker activity, especially against Gram-negative bacteria.

As mentioned above, the cell wall of Gram-positive bacteria contains teichoic acids. They not only have structures with a negative charge, but also fragments with a positive charge such as amino groups. It is with them that anionic surfactants bind, which often leads to disruption of the cell wall and increased permeability. These compounds can also interact with proteins responsible for the biosynthesis of the peptidoglycan contained in the cell wall, damage transport and enzyme proteins, and affect the energy capacity of the cell by affecting ATP-ases. In the cell of Gram-negative bacteria, anionic surfactants interact with lipopolysaccharides (LPSs) contained in the cell wall, specifically with their amphoteric fragments. This leads to the destabilization of the inner membrane and an increase in its permeability. The outer membrane also contains porin proteins, the configuration and function of which may be altered by the action of anionic surfactants, resulting in the opening of transport channels. Changes in the structure of proteins contained in the outer structures of the cell also affect the mechanisms of adhesion, stress response or biofilm production. Anionic surfactants also disrupt ionic equilibrium and contribute to the release of lytic enzymes, and consequently, damage to DNA structures can be observed. These compounds can also induce oxidative stress, which further contributes to cell death [44,45,46].

The combination of the action of cationic compounds with anionic ones may produce a synergistic effect and enhance the antimicrobial action, but it may also be weakened by the mutual suppression of the effect of the charges. In addition to the influence of the surfactant charge itself, antimicrobial activity also depends on other components such as the number and length of hydrocarbon chains, the structure of the hydrophilic component, the so-called head, and the number of repeated hydrophilic and hydrophobic segments. Nowadays, an increasing number of publications deal with gemini surfactants, which possess a broad spectrum of biocidal activity [32,35,39,47].

All obtained series of catanionic surfactant mixtures were subjected to tests to determine the activity against Gram-positive bacteria, *Staphylococuc aureus*, and Gram-negative bacteria, *Escherichia coli*. The obtained values of the minimum inhibitory concentration are listed in Table 2 and Table 3 for double and single catanionic mixtures, respectively.

**Figure 2:**
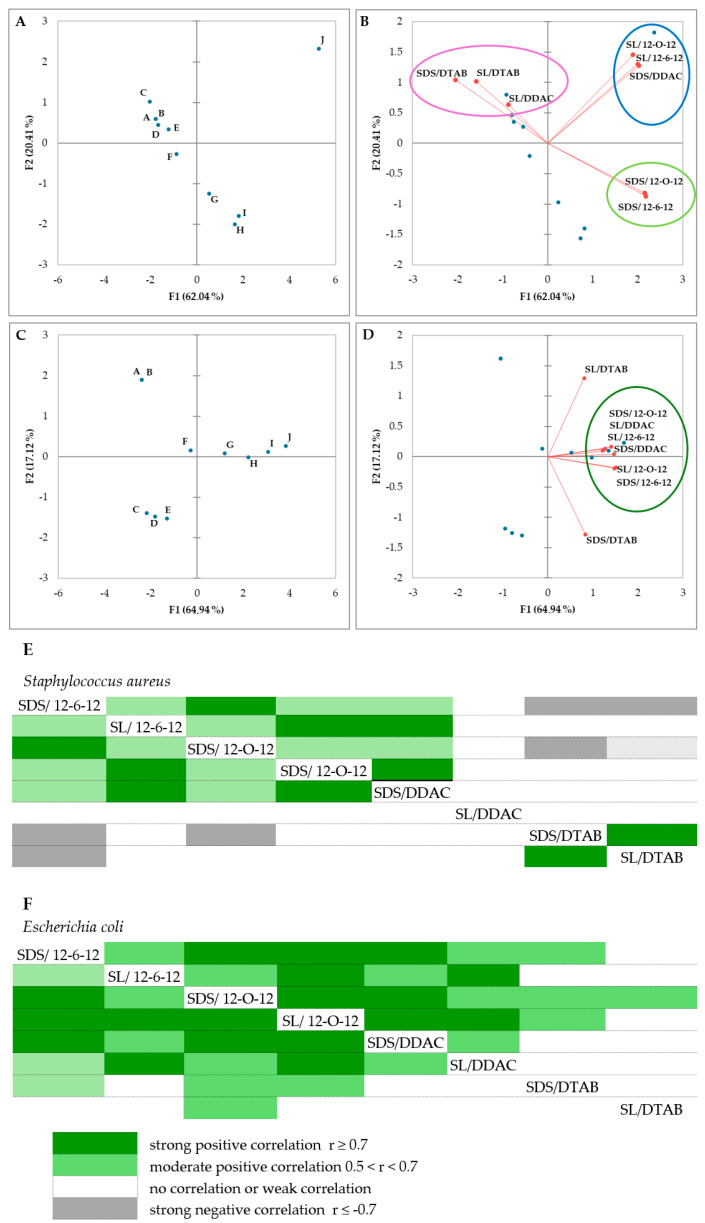
Principal component analysis for the minimum inhibitory concentrations (MICs) of eight substances in ten variants for *Staphylococcus aureus* (**A**,**B**) and *Escherichia coli* (**C**,**D**). Circles for subfigures (**B**,**D**) show groupings of compounds with similar relationships. Composition variants are shown as blue dots, while substances as red lines with red dots. (**E**,**F**) Correlation matrix for mixtures of surfactants (MICs) for *Staphylococcus aureus* and *Escherichia coli*, respectively.

A biplot via PCA indicated the configuration of MIC distributions of eight surfactant mixtures in 10 different ratios for *S. aureus*, as shown in Figure 2A,B. The directions of the vectors shown in the graph indicate whether they are positively or negatively correlated. All compounds, for Gram-positive bacteria, were divided into three sets. In each, the mixtures tested are positively correlated with each other, meaning that the sensitivity of cells to these mixtures may be similar.

Correlation matrix analysis showed that MICs of mixtures containing (a) gemini surfactants and SDS and (b) gemini surfactants and SL, as well as the monomeric surfactant DDAC in a mixture with SDS, are strongly positively correlated (r > 0.9), indicating a more similar mechanism of action on *S. aureus* compared to the other compounds. In a comparison of SDS/12-O-12 and SDS/12-6-12 samples with compounds containing the monomeric surfactants SDS/DTAB and SL/DTAB, on the other hand, strong negative correlations (r > 0.7) are evident (Figure 2E). Comparing the MIC results for these cases (Table 2 and Table 3), it can be seen that as the concentration of anionic compounds increases for the first group—containing gemini surfactants—the values increase, and for the second group, they decrease.

In the case of *E. coli*, only mixtures containing DTAB in their composition are outside the common set. The other vectors corresponding to individual surfactant mixtures lie close to each other on the biplot (Figure 2C,D) and are positively correlated. This means that the variables change in the same direction in the principal component space. In a comparison of individual compound pairs with each other, a strong positive correlation (r > 0.8, Figure 2F) is also evident in many cases.

The antibacterial properties of the catanionic mixtures have been determined as a function of the mixing ratio. As a general trend, the antibacterial activity increases with an increase in the cationic charge density in the mixtures. Our results are in agreement with those described by Perez et al. [48] who reported that the antibacterial activity of the catanionic mixtures depends on both the proportion of cationic surfactant and the microorganism tested. The results (Table 2 and Table 3 and Figure 2) indicate that the bactericidal activity of the catanionic mixtures resulted in a lack of synergism with respect to the surfactant alone. With the increase in the concentration of anionic surfactant, the MIC values increase, i.e., the antibacterial activity decreases. This suggests that the activity of these systems is governed by the concentration of cationic compounds. For the rich anionic mixture, the positive charge of the cationic surfactant is totally neutralized, and the MIC values increase.

### 2.3. Quantum Mechanical Calculations

An attempt to link the antibacterial activity with the structure of the studied systems was made based on the inquiry of frontier molecular orbitals. Analysis of the HOMO and LUMO orbitals can provide valuable information on the reactivity of molecules and possible charge transfer interactions. These properties are characterized by the energy ΔE_gap_, equal to the difference in the orbital energies E_LUMO_ and E_HOMO_ (ΔE_gap_ = E_LUMO_ − E_HOMO_) (Table 4). The smaller the energy gap, the higher the probability of the charge transfer process [49,50]. Thus, high reactivity is associated with a small HOMO-LUMO gap. An attempt was made to estimate the reactivity of the studied three systems: (1) containing only a cationic surfactant molecule, (2) a cationic surfactant molecule with an anionic surfactant molecule (SDS or SL) and (3) a cationic surfactant molecule and two SDS or SL molecules, respectively.

**Figure 3:**
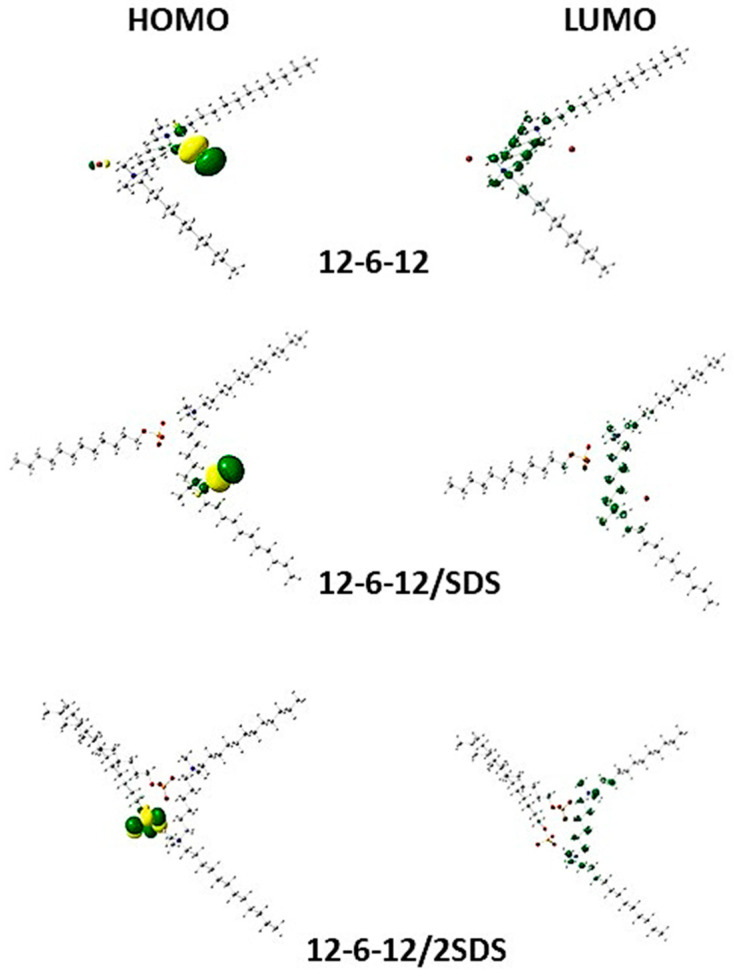
Frontier molecular orbitals of 12-6-12, 12-6-12/SDS and 12-6-12/2SDS (white—hydrogen, grey—carbon, blue—nitrogen, green/yellow—bromide, red—oxygen, orange—sulphur).

For 12-6-12, it was observed that the ΔE_gap_ values are almost equal when moving from a surfactant molecule alone to a system containing one 12-6-12 molecule and one SDS molecule. When adding a second SDS molecule, the ΔE_gap_ value grows significantly. A similar trend was detected for the 12-O-12 system with SDS. In the case of systems 12-6-12 and 12-O-12 to which the anionic surfactant SL was added, the differences in ΔEgap values are small. For DTAB and DDAC, there is an increase in the ΔE_gap_ value after the addition of the first molecule of anionic surfactant SDS to the studied systems. Frontier molecular orbitals calculated for 12-6-12 and other systems are shown in Figure 3. In the 12-6-12 set, the HOMO is distributed mainly on the quaternary nitrogen atom, the bromine anion, or the head of SDS, while the LUMO is primarily dispersed over the spacer of the cationic surfactant.

**Figure 4:**
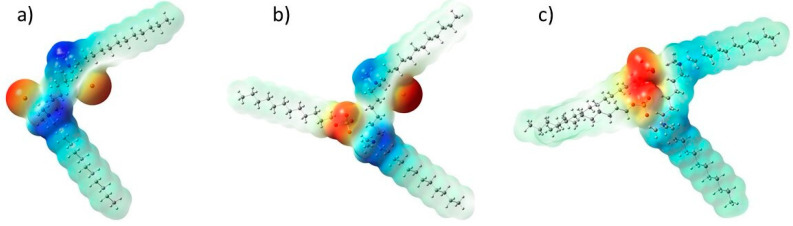
Molecular electrostatic potential maps for (**a**) 12-6-12, (**b**) 12-6-12/SDS and (**c**) 12-6-12/2SDS.

The electrostatic potential generated in the space around a molecule can be analyzed to predict the reactive behavior of molecules [51]. The electrostatic potential maps were calculated for the surfactant 12-6-12 and the systems containing 12-6-12 and one or two SDS molecules (Figure 4). Colors reflect areas of different values of electrostatic potential. The area marked in red is characteristic of the most electronegative molecular fragments, while the most electropositive are represented by blue color. In this respect, the blue space around the quaternary nitrogen atoms in 12-6-12 stands out, which may be important in the context of the interaction of the surfactant molecule with bacterial cell membranes. As SDS molecules are added and interact with the surfactant molecules, the disappearance of the blue color around the quaternary nitrogen atoms and along the six-carbon linker is observed, which is associated with decreasing electrophilic properties of the system.

## 3. Materials and Methods

### 3.1. Materials

Dodecyldimethylammonium chloride (99%), dodecyltrimethylammonium bromide (98%), sodium dodecyl sulfate (97%) and sodium dodecanoate (99%) were obtained from Merck (Poznan, Poland). Gemini surfactants were obtained and analyzed according to the procedure developed in our laboratory, [11] for 12-6-12 and [39] for 12-O-12.

### 3.2. Antimicrobial Activity

The catanionic mixtures were tested for antimicrobial activity against bacteria: *Escherichia coli* ATCC 10536 and *Staphylococcus aureus* ATCC 6538. The MIC values for all microorganisms were determined by a tube standard two-fold dilution method [52]. Each of the microorganisms was resuspended in a physiological salt solution and diluted to 10^7^ cfu/mL. In the next step, 1 mL of microorganism suspension was mixed with 1 mL of media: TSB (MERCK, Darmstadt, Germany) containing serial dilutions of the tested mixtures. All samples were incubated at 37 °C for 24 h. As a growth control, a suspension of microorganisms in a medium without the biocides was used. The MICs were defined as the lowest concentration of the mixtures in which there was no visible growth. All tests were repeated three times.

The principal component analysis (PCA) for the MIC value of the eight different substances was performed using XLSTAT (version 2021.5, Lumivero, Denver, CO, USA). *p* < 0.05 was considered statistically significant.

### 3.3. Computational Quantum Mechanical Modeling Method

DFT calculations were performed using the GAUSSIAN16 [53] program package with the APF-D functional [54,55] and the 6-311++g(d,p) basis set [56]. The APF-D functional was chosen because it provides an excellent compromise between accuracy and computational cost. In the first step, geometry optimization was performed for 12-6-12, 12-O-12, DDAC and DTAB. Then, the optimized geometry of these cationic surfactants was frozen during optimization with the addition of one and two molecules of SDS or SL, respectively. The calculations were performed for molecules surrounded by water, which was achieved using the conductive screening solvation model. In this model, the solvent is treated as a structureless dielectric continuum [57]. The HOMO and LUMO energy values from a.u. were converted to eV (1 a.u. = 27.2114 eV).

## 4. Conclusions

We obtained eight series of catanionic surfactant mixtures in different cationic/anionic molar ratios. The synergism observed in the case of surface properties of cationic mixtures is not observed in the case of antimicrobial activity. The addition of anionic additives was found to adversely affect the antibacterial activity in the case of both Gram(+) and Gram(−) bacteria of cationic ammonium microbicides. With a small addition of anionic surfactant to gemini surfactants, antimicrobial activity is preserved. This conclusion is of great importance from an economic point of view, due to the possibility of reducing microbial populations on an industrial scale by creating surfactant compositions. Quantum mechanical tests confirm that the anionic additive reduces the behavior of molecules, which is in good correlation with experimental microbiological data.

## Figures and Tables

**Table 1:** Amounts of particular cationic (A1) and anionic (B1) surfactants used to create catanionic mixtures (the initial surfactant concentration was 1 mM).

No.	Volume of Solutions [mL]			Molar Ratio
	A1 ^1^	B1 ^2^	H_2_O	A1/B1
0	25	0	75	-
1/A	25	1	74	25
2/B	25	2	73	12.5
3/C	25	5	70	5
4/D	25	10	65	2.5
5/E	25	20	55	1.25
6/F	25	30	45	0.83
7/G	25	40	35	0.625
8/H	25	50	25	0.50
9/I	25	60	15	0.42
10/J	25	75	0	0.33

^1^ 12-6-12, 12-O-12, DTAB or DDAC, ^2^ SDS or SL.

**Table 2:** MIC values (mM) for gemini catanionic mixtures with increasing amount of anionic surfactant.

No.	SDS/12-6-12		SL/12-6-12		SDS/12-O-12		SL/12-O-12	
	*S. aureus*	*E. coli*	*S. aureus*	*E. coli*	*S. aureus*	*E. coli*	*S. aureus*	*E. coli*
0	0.007813	0.03125	0.007813	0.03125	0.007813	0.03125	0.007813	0.03125
1/A	0.007813	0.007813	0.007813	0.015625	0.007813	0.03125	0.007813	0.015625
2/B	0.007813	0.007813	0.007813	0.015625	0.007813	0.03125	0.007813	0.015625
3/C	0.007813	0.015625	0.007813	0.015625	0.015625	0.03125	0.007813	0.015625
4/D	0.007813	0.015625	0.007813	0.015625	0.015625	0.03125	0.007813	0.03125
5/E	0.015625	0.03125	0.007813	0.015625	0.015625	0.03125	0.007813	0.03125
6/F	0.015625	0.03125	0.007813	0.015625	0.015625	0.0625	0.007813	0.03125
7/G	0.03125	0.0625	0.007813	0.015625	0.03125	>0.0625	0.007813	0.03125
8/H	>0.0625	>0.0625	0.007813	0.03125	0.0625	>0.0625	0.007813	0.0625
9/I	>0.0625	>0.0625	0.015625	0.03125	>0.0625	>0.0625	0.007813	0.0625
10/J	>0.0625	>0.0625	0.0625	>0.0625	>0.0625	>0.0625	0.03125	>0.0625

**Table 3:** MIC values (mM) for monomeric ammonium catanionic mixtures with increasing amount of anionic surfactant.

No.	SDS/DDAC		SL/DDAC		SDS/DTAB		SL/DTAB	
	*S. aureus*	*E. coli*	*S. aureus*	*E. coli*	*S. aureus*	*E. coli*	*S. aureus*	*E. coli*
0	0.009766	0.039063	0.009766	0.039063	0.15625	0.3125	0.15625	0.3125
1/A	0.009766	0.039063	0.009766	0.039063	0.15625	0.3125	0.15625	0.625
2/B	0.009766	0.039063	0.009766	0.039063	0.15625	0.3125	0.15625	0.625
3/C	0.009766	0.039063	0.019531	0.039063	0.15625	>0.625	0.15625	0.3125
4/D	0.004883	0.039063	0.009766	0.039063	0.15625	>0.625	0.15625	0.3125
5/E	0.019531	0.15625	0.019531	0.078125	0.15625	>0.625	0.078125	0.3125
6/F	0.009766	0.039063	0.009766	0.039063	0.15625	>0.625	0.078125	0.625
7/G	0.019531	0.625	0.009766	0.078125	0.078125	>0.625	0.078125	0.625
8/H	0.019531	>0.625	0.009766	0.039063	0.078125	>0.625	0.078125	>0.625
9/I	0.019531	>0.625	0.009766	>0.625	0.078125	>0.625	0.078125	>0.625
10/J	0.15625	>0.625	0.009766	>0.625	0.078125	>0.625	0.078125	>0.625

**Table 4:** Values of E_LUMO_, E_HOMO_ and ΔE_gap_ of cationic surfactants and their mixtures with SDS and SL.

Mixture	E_HOMO_ [eV]	E_LUMO_ [eV]	ΔE_gap_ [eV]
12-6-12	−6.5721	−0.1355	6.4366
12-6-12/SDS	−6.5650	−0.1268	6.4382
12-6-12/2SDS	−7.3441	−0.1020	7.2420
12-6-12/SL	−6.3631	−0.1268	6.2363
12-6-12/2SL	−6.3128	−0.0952	6.2175
12-O-12	−6.6418	−0.1461	6.4956
12-O-12/SDS	−6.6208	−0.1298	6.4910
12-O-12/2SDS	−7.4225	−0.1094	7.3131
12-O-12/SL	−6.4162	−0.1314	6.2847
12-O-12/2SL	−6.3729	−0.1238	6.2491
DDAC	−6.8790	−0.0566	6.8224
DDAC/SDS	−7.4777	−0.0463	7.4314
DDAC/2SDS	−7.4366	−0.1902	7.2464
DDAC/SL	−6.2970	−0.0906	6.2064
DDAC/2SL	−6.2769	−0.1902	6.0866
DTAB	−4.9987	−0.9129	4.0858
DTAB/SDS	−6.2788	−0.7644	5.5144
DTAB/2SDS	−7.1465	−0.4874	6.6592
DTAB/SL	−5.4806	−0.6370	4.8436
DTAB/2SL	−5.3759	−1.2969	4.0790

ΔE_gap_ = E_LUMO_ − E_HOMO_.

## Data Availability

The data presented in this study are available in this article.
